# Biosensors based on lithotrophic microbial fuel cells in relation to heterotrophic counterparts: research progress, challenges, and opportunities

**DOI:** 10.3934/microbiol.2018.3.567

**Published:** 2018-07-20

**Authors:** Hai The Pham

**Affiliations:** 1 Research group for Physiology and Applications of Microorganisms (PHAM group), GREENLAB, Center for Life Science Research, Faculty of Biology, Vietnam National University-University of Science, Nguyen Trai 334, Thanh Xuan, Hanoi, Vietnam; 2 Department of Microbiology, Faculty of Biology, Vietnam National University-University of Science, Nguyen Trai 334, Thanh Xuan, Hanoi, Vietnam

**Keywords:** microbial fuel cell, bioelectrochemical systems, iron bacteria, iron oxidation, lithotrophic biosensor

## Abstract

Biosensors based on the microbial fuel cell (MFC) platform have been receiving increasing attention from researchers owing to their unique properties. The lithotrophic MFC, operated with a neutrophilic iron-oxidizing bacterial community, has recently been developed and proposed to be used as a biosensor to detect iron, and likely metals in general, in water samples. Therefore, in this review, important aspects of the lithotrophic MFC-based biosensor, including its configuration, fabrication, microbiology, electron transfer mechanism, sensing performance, etc. were carefully discussed in comparison with those of heterotrophic (organotrophic) counterparts. Particularly, the challenges for the realization of the practical application of the device were determined. Furthermore, the application potentials of the device were also considered and positioned in the context of technologies for metal monitoring and bioremediation.

## 1. Introduction

Biosensors are defined as devices that employ biological elements for sensing analytes to produce signals through their interactions with transducers [1]. Biological sensing elements can include enzymes, nucleic acids, antibodies or antigens, receptors, and microorganisms, as well as animal and plant cells and even whole organisms [1],[2]. Among these elements, microorganisms appear to be more advantageous because: (i) they can detect a wide range of chemical substances, (ii) they are amenable to genetic modification, and (iii) they can operate in broad pH and temperature ranges. Furthermore, due to their microscopic size and high growth rate, they offer rapid and relatively precise responses to changes of the analytes to be sensed. Microorganisms have thus been used in a variety of biosensing systems with various types of signal transducing methods, including amperometric, potentiometric, calorimetric, conductimetric, colorimetric, luminescence-based, and fluorescence-based sensors [2]. Numerous publications and reviews have discussed those types of systems [1],[2]. Recently, the emergence of microbial fuel cell technology has given rise to novel biosensors based upon microbial fuel cells [3]–[8], which offer a number of promising application potentials in practice, such as monitoring biological oxygen demand (BOD), detection of toxic metals and compounds, warning of dramatic changes of water treatment system inflows and outflows, monitoring microbial activities, and biofilm corrosion, etc. Microbial fuel cells (MFCs) are bioelectrochemical systems that operate with microorganisms acting as catalysts to convert chemical energy to electrical energy [9]. Although the possibility of using MFCs for harnessing power from waste appears very appealing, the technology has not been applied in reality due to the low maximum power output (per volume unit) of a MFC [10],[11]. That is why other application potentials of MFCs, including the development of novel biosensors, have recently received more research attention [12]–[17]. In comparison to other types of microbial sensors, MFC-based biosensors offer advantages in that they can be self-powered and that they can produce electrical signals reflective of the metabolic rate of microorganisms, which can indicate the level of metabolized substrates. Moreover, MFCs directly generate electrical signals, eliminating the need for separate transducers, which is essential in other microbial sensor systems.

Most MFC-based or bioelectrochemical (BES)-based biosensors reported thus far are operated with organic substrates as fuel, or electron donors [18],[19]. These substrates are mostly artificial or real wastewater containing mixtures of organic compounds [18],[19]. Thus, the sensing mechanism of those biosensors, whether to monitor the concentration of the analyte or to detect toxicity, is based on the microbial metabolism of organic matters. Indeed, a number of studies on those sensing systems have been reported and thoroughly reviewed [14],[19]. In contrast, inorganic electron donors have been rarely examined in MFC-based sensors. Only recently have several MFC (or BES) systems operated with inorganic electron donors (substrates), such as sulfide, reduced inorganic sulfur compounds, ammonium, or metal ions, e.g. Fe^2+^, have been reported [20]–[24]. However, regarding their use as biosensors, only those operated with metal ions as electron donors have been studied [17],[23]. These systems, in which electrons were generated by the chemolithotrophic metabolism of the anodic bacteria, should be designated as lithotrophic MFC-based biosensors. As these systems have only emerged recently, an overview of the current research in relation to that of their counterparts may be helpful. Therefore, this paper reviews ongoing research on lithotrophic MFC-based biosensors while comparing them with heterotrophic systems and extending the discussion on the challenges and opportunities for contextual application.

## 2. Current discoveries

Firstly, it should be noted that, as mentioned above, there has been a long history of extensive study on MFC-based biosensors, mostly involving those operated with heterotrophic microorganisms. Therefore, current discoveries of the lithotrophic MFC-based biosensor should be viewed in a comprehensive comparison with previous findings on these heterotrophic counterparts (Table 1).

### 2.1. Device configuration and fabrication

The first lithotrophic MFC-based biosensor proposed by Nguyen et al. (2015) was fabricated following the National Centre for Biotechnology Education (UK) (NCBE) model—a conventional design [23]. Basic materials for MFC assembly [25] were used, including polyacrylic as the frame material, graphite granules as the electrode material, and Nafion as the cation-exchanging membrane. The electrical signals can be monitored upon a voltage-based or a current-based manner. With such a design, the device was operated in batch mode, which provided for its function to sense Fe^2+^ and Mn^2+^ in water samples. Nevertheless, a special feeding scheme with a separate supply of the sample containing the metal ions was required in order for the device to work efficiently [23]. As the research on lithotrophic MFC-based biosensors is currently in an elementary phase, no further modified designs have been tested.

It should be noted that several designs for MFC typed biosensors have been previously proposed. These include also a modified two-chamber NCBE-type design applied for a toxicity sensor [26]. The optimized two-chamber configuration and the so-called sensor-type configuration proposed by Kim et al. may enable a more responsive BOD sensor with less operational variations [27],[28]. The air cathode or single chamber configuration, which was developed later, offers a more compact device with simpler operation and thus lower operational cost [4],[29]. Recent attempts to create even more compact sensors eventually led to the development of microsized sensors [6],[30],[31]. The size and configuration of the latter were engineered so as to increase their response sensitivity and accuracy in addition to their practical application feasibility. It is therefore essential that innovative designs should also be included in experimentation for lithotrophic MFC-based biosensors in order to improve their performance.

MFC-based biosensors can be operated in batch mode [29], but in most cases, they are operated in continuous mode, which is recommended to obtain more reliable signals and more importantly, to enable on-line monitoring [3],[5],[32]. Therefore, operating lithotrophic MFC-based biosensors in continuous mode may be a promising approach to enhance their sensing capacity.

### 2.2. Electron donor

The lithotrophic MFC-based biosensor proposed by Nguyen et al. (2015) is able to utilize ferrous iron as the electron donor [23]. The MFC generated an electrical current when it was operated with FeCl_2_ as the only possible electron donor at the anode and HCO_3_^−^ as the carbon source [23]. It was later found that Mn^2+^ could also serve as an electron donor for such type of MFC, but the device did not seem to function well with a concentration of Mn^2+^ above 3 mM [17]. In addition, the presence of Ni^2+^ and Pb^2+^ might inhibit the electricity generation coupled with iron oxidation of the MFC. Organic electron donors were also tested in the MFC and did not appear to be the favored substrates [17]. Thus, Tran et al. (2015) claimed that the microorganisms in the MFC system might be so specialized in lithotrophy that their most favorable electron donors are metal ions.

**Table 1.** A comprehensive comparison of the lithotrophic MFC-based biosensor and the heterotrophic counterparts.

Compared factor	Lithotrophic MFC-based biosensor	Heterotrophic MFC-based biosensor
Analyte	Fe^2+^ (Mn^2+^)	BOD, toxicity
Electron donor	Fe^2+^ (Mn^2+^)	Organic compounds
Configuration	Modified NCBE type (two-chamber, square shape)	Sensor-type [27],[28]; single-chamber [4],[29]; microsized reactor [6],[30],[31]
Operational mode	Batch	Continuous
Working microorganisms	Bacterial community dominated by neutrophilic *Pseudomonas* sp. that possibly oxidize Fe^2+^	Various heterotrophic communities
Possible electron transfer mechanism	Indirect, via self-secreted mediators	Various mechanisms: direct and indirect electrode interactions
Sensing performance:		
Shortest response time	1 min	3 min
Detection range	3–20 mM for Fe^2+^; 1–3 mM for Mn^2+^	3–150 mg L^−1^ for BOD [3],[26],[27]; 0–350 mg L^−1^ for COD [30]; varying toxic compounds (lowest concentration detected: 1 µg L^−1^ for cadmium [30])
Specificity	High	Low; Acclimatized systems have high specificities [33],[34]
Challenges	Narrow detection range Performance stability Proper inoculation source	Limited specific detection Performance stability
References	[17],[23]	[3],[4],[6],[27],[28]–[34]

Indeed, MFCs operated with other inorganic electron donors have been also reported (Table 2). Rabaey et al. (2006) reported a MFC system operated with sulfide as an inorganic electron donor, but only able to be utilized in the presence of another organic electron donor, such as acetate [24]. Thus, this type of MFC is not a fully lithotrophic MFC. Interestingly, in a more recent study Zhong et al. (2017) reported a fully lithotrophic system operated with sulfide as the sole electron donor and nitrate as the electron acceptor [21]. Furthermore, other fully lithotrophic MFCs were also proven to be feasibly functional with inorganic sulfur compounds, such as tetrathionate, as the electron donors and ferric iron as the electron acceptor [20],[35]. In another context, He et al. (2009) reported the development of a MFC with a rotating cathode that efficiently generated electricity while ammonium salts were present in the anolyte [22]. As those systems were not developed for use as biosensors, they will not be discussed further in this review.

In heterotrophic MFC-based biosensors electron donors are, by default, organic compounds, which are actually BOD contents in wastewater in most cases [4],[27]. In some laboratory-scale systems acetate, a specific volatile fatty acid, or a mixture of glucose and glutamate may be used [3],[33],[34],[36]. These organic electron donors are thermodynamically favored by anode bacteria, as they provide more energy than inorganic electron donors. Nevertheless, as they are organic in nature, the biosensors can solely be used to sense BOD changes or toxicity, indicating that their sensing selectivity is limited. However, in some cases when proper acclimatization of the anode bacteria was achieved, the sensing was able reach certain specificity levels [33],[34].

**Table 2.** Some MFC systems operated with inorganic electron donors.

MFC type	Electron donor	Configuration	Electricity generation	Operated as biosensor	Reference
Sulfide removing microbial fuel cell	Sulfide coupled to acetate	Square-type (modified two-chamber NCBE type) and tubular type	Maximum current: 11 mA	No	[24]
			Power output: 37 mW L^−1^ NAC (20 mW L^−1^ TAC)		
Denitrifying sulfide removing microbial fuel cell	Sulfide only	Modified two-chamber NCBE type	Maximum current: NA	No	[21]
			Power output: 2.80 ± 0.05 mW L^−1^ NAC		
Reduced inorganic sulfur compound (RISC) removing microbial fuel cell	RISC (tetrathionate)	Modified two-chamber NCBE type	Maximum current: 0.39 mA*	No	[20],[35]
			Power output: 1.7 mW L^−1^ TAC*		
Ammonia removing microbial fuel cell	Ammonium	Rotating-cathode type	Maximum current: 0.078 ± 0.003 mA	No	[22]
			Power output: NA		
Iron-oxidizing microbial fuel cell (lithotrophic MFC)	Ferrous iron (Fe^2+^); Manganese (II) (Mn^2+^)	Modified two-chamber NCBE type	Maximum current: 0.6 mA	Yes	[17],[23]
			Power output: 2.56 mW L^−1^ NAC		

NAC: net anodic compartment; TAC: total anodic compartment; NA: not available; *: recalculated from the authors' calculation.

### 2.3. Microbiology

The role of the microbial source: Nguyen et al. (2015) proved that a microbial source used for inoculation is necessary to enrich a well- and stably-performing working bacterial community that can oxidize iron and produce electricity in a MFC [23]. According to them, the inoculum for enrichment should be taken from sites where clear indicators of metal metabolism are evident. After enrichment, the established reactor (the so-called lithotrophic MFC) is to be operated as an open system. In such a system, the mixed culture community, which is enriched from a natural microbial source, seems to enable a stable, long-term performance of the MFC [37],[38]. Indeed, similar results from inoculation have been reported elsewhere, even in heterotrophic systems [39],[40]. There was clear evidence that the inoculum selection could significantly affect the performance of MFCs in general and MFC-typed biosensors in particular [41]–[43].

Microbial communities and typical microorganisms: By DNA analysis and fluorescent in situ hybridization (FISH) analysis, Nguyen et al. (2015) showed that bacteria in the anode compartment of a lithotrophic MFC-based biosensor were solely present in the suspension and did not tend to form a noticeable biofilm on the electrode surface [23]. Particularly, a clear increased presence of neutrophilic, iron-oxidizing bacteria was observed at the anode of the MFC, which suggests that the iron oxidation coupled to electricity generation of the MFC is likely linked to the activity of these bacteria [23]. Interestingly, *Geobacter* sp., *Bacillus* sp., and specifically *Pseudomonas* sp. were found to be dominant in the community. These bacteria, with the exception of *Bacillus* sp., have been discovered in many other bioelectrochemical systems and are considered key players in the electrochemical activity of the systems [9],[44]. Thus, they are also believed to play important roles in the performance of the lithotrophic MFC, as “neutrophilic iron bacteria” [23]. Therefore, from the first glance, one may suppose that the microbial community of a lithotrophic MFC may have a high similarity to those of other MFC systems (including heterotrophic systems), but it actually does not, considering the possibility that the electrochemically active bacteria in a lithotrophic MFC can be so specialized in their lithotrophic metal-oxidizing capabilities. This possibility is due to the fact that Fe^2+^ was the favored substrate over organic electron donors such as acetate, lactate, or BOD materials (when present at non-excessive levels) [17]. Thus, it was suggested that the bacteria at the anode of the lithotrophic MFC may be novel species that are not well understood, or they are known species with unknown activities.

### 2.4. Proposed electron transfer mechanisms

**Figure 1.**
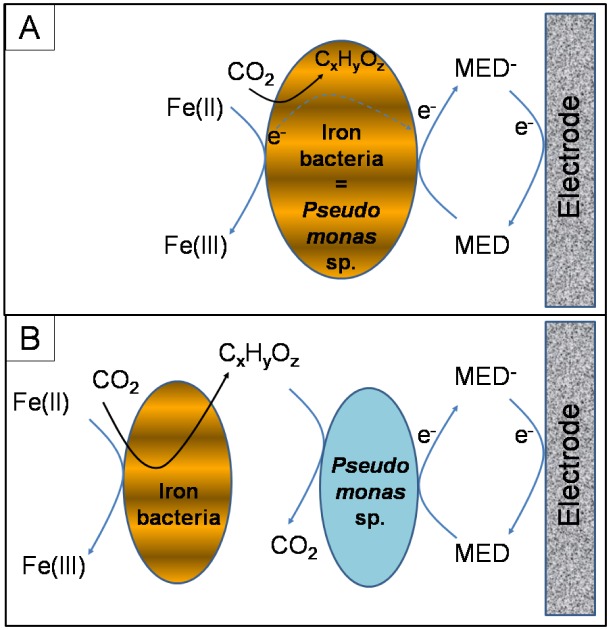
The main hypothesis (A) and the alternative hypothesis (B) of electron transfer mechanism at the anode of the iron-oxidizing lithotrophic MFC (partially adopted from [23]). Notes: MED: mediator.

It was found that iron-oxidizing bacteria are present in the anode microbial community of the lithotrophic MFC but they did not tend to form a noticeable biofilm on the electrode, which leads to a hypothesis that those bacteria are involved in the anode electron transfer but they do not directly transfer electrons to the electrode [23]. Furthermore, the dominance of *Pseudomonas* species in the anode community of the MFC also supports this hypothesis. *Pseudomonas* species are well known as electrochemically active heterotrophic bacteria that can self-produce electron mediators to reduce an electrode through oxidization of organic matter in heterotrophic MFCs [45],[46]. Several *Pseudomonas* species have been also reported to be able to chemolithotrophically metabolize Fe^2+^ at neutral pH [47],[48]. Therefore, considering the facts mentioned above, Nguyen et al. (2015) hypothesized that some *Pseudomonas* species could be actually the dominant “neutrophilic iron bacteria” in the anode of the lithotrophic MFC and can oxidize Fe^2+^, as well as transfer electrons to the anodic electrode via their self-produced mediators (Figure 1) [23]. Thus, in a lithotrophic MFC-based biosensor, the anode electron transfer is believed to be based on the indirect mechanism [45]. This mechanism is not new, but it should be noted that the self-mediated pseudomonads may also utilize an inorganic electron donor, such as Fe^2+^, to harvest energy- they are electrochemically active lithotrophs.

Although the above-mentioned hypothesis of electron transfer mechanism is strongly supported by experimental data, one should not neglect a possibility that pseudomonads may utilize organic electron donors synthesized from the iron chemilithotrophy of certain neutrophilic iron oxidizing bacteria, or even “graze” on their cells (biomass). This is somewhat supported by our recent observation that after a significant period of operation (e.g. a year), the lithotrophic MFC could generate electrical current for a short time when no ferrous iron was supplied (unpublished data). Furthermore, the amount of energy harvested from the oxidation of ferrous iron at neutral pH is considerably too thermodynamically small to compensate for the expense of self-producing mediators. Therefore, some alternative electron transfer mechanisms for the anode reaction of the lithotrophic MFC-based biosensor may be hypothesized (Figure 1).

### 2.5. Sensing performance

#### 2.5.1. The capability of the lithotrophic MFC as a sensor for iron and manganese

The lithotrophic MFC, once enriched with iron-oxidizing bacteria, could be operated with anode solutions containing Fe^2+^ and having a pH 7 [23]. As reported by Tran et al. (2015), when operated with different concentrations of Fe^2+^ in the anode influent, the lithotrophic MFC generated electrical current and charge that were proportional to the concentration of Fe^2+^ from 5 mM to 20 mM (r^2^ = 0.98) [17]. This response was the same no matter if the concentrations of Fe^2+^ were tested in increasing or decreasing concentrations. Experimental results also showed that the MFC generated current only when Fe^2+^ was present in the anode buffer and that the generation of current is mostly due to biotic oxidation of Fe^2+^ [17]. Based on those findings, the authors suggested that the MFC could be used as a biosensor to detect iron, or even to measure the amount of ferrous iron (within a range) in a water sample having a neutral pH. Similar responses were also observed when the lithotrophic MFC was operated with Mn^2+^, but the range of Mn^2+^ concentration to which the MFC responded was limited to less than 3 mM. Thus, the capability of the MFC as a biosensor for manganese does not seem to be as efficient as that seen for iron. Tran et al. (2015) hypothesized that inhibitory effects of Mn^2+^ on the anode microorganisms might be the reason for the decreased performance.

#### 2.5.2. Sensitivity and detection limits

The response time of the MFC (i.e. time for the current to reach a steady state in any test) was approximately 60 sec when the concentration of Fe^2+^ was step increased, while it took a period of one batch run (ca. 2 hrs) when the concentration of Fe^2+^ was step decreased. Thus Tran et al. (2015) claimed that the lithotrophic MFC-based biosensor has a relatively high sensitivity when sensing increasing concentrations of Fe^2+^, but its sensitivity may be reduced if decreasing concentrations are to be monitored [17]. By testing the concentrations of Fe^2+^ from 1 to 5 mM, Tran et al. (2015) also determined that the lower detection limit of the lithotrophic MFC-based biosensor for Fe^2+^ was 3 mM. Furthermore, as the device did not respond to changes in Fe^2+^ concentration exceeding 20 mM—this concentration was determined as the upper detection limit. As for manganese, although the lower detection limit of the device was about 1 mM, the upper detection limit was determined to be only 3 mM, indicating that the anode bacteria might be more susceptible to Mn^2+^.

#### 2.5.3. Endurance

It was shown that the lithotrophic MFC could endure starvation (being operated without feeding) for a period of up to 14 days and fully restore its capability of generating electricity after starvation [17]. On the other hand, the response of the system to changes in the concentration of Fe^2+^ remained unchanged after 12 months of operation, although a generated current reduced by 25% could be observed. Therefore, the lithotrophic MFC-based iron sensor can be considered to have a stable performance and thus can be used for long term application.

#### 2.5.4. Specificity

It should be noted that some metal ions, such as Ni^2+^ and Pb^2+^, could act as possible inhibitors on the anodic microorganisms in the lithotrophic MFC-based biosensor but not as competing electron donors [17]. This finding suggests that among metals, the device responds more specifically to iron and manganese. In fact, the anodic microorganisms in the MFC could use organic compounds such as acetate, lactate, or glucose/glutamate mixtures as electron donors [17]. However, it is remarkable that these organic electron donors, if not present at excessive levels, were not favored over Fe^2+^. Thus, it was concluded that, when co-present with Fe^2+^ in the anode, organic compounds did not interfere with the response of the MFC to the presence of Fe^2+^. Altogether, the current research suggests that the lithotrophic MFC-based biosensor has a remarkably high specificity to iron.

Regarding heterotrophic MFC-based systems, as the result of extensive study thus far, their sensing performances have been seemingly much more optimized. As mentioned earlier, these systems can be used most efficiently to monitor BOD and toxicity of wastewater. Thus, specificity is not a serious requirement for these devices, although selective detection of certain organic compounds could be achieved through proper acclimatization of the anode bacterial communities [33],[34]. With respect to detection range, the first systems are able to respond well to BOD concentrations ranging from 3–150 mg L^−1^ [3],[26],[27] and the upper limit was later even improved up to 350 mg L^−1^ with a single chamber configuration [30]. The systems could also detect a variety of toxicants including phenolic or halogenated compounds and some heavy metals [5],[6],[30],[32] with a high sensitivity, particularly when the MFC was miniaturized. According to di Lorenzo et al. (2014) [30], a small-scale MFC-based biosensor could detect cadmium at a concentration as low as 1 µg L^−1^ with a detection time of 3 min. It can be noted that the detection range and sensitivity of the heterotrophic systems are considerably suitable for practical application [19], which is an advantage over the lithotrophic counterparts, although the research of which is still in an early phase.

### 2.6. Effect of operational parameters

When applying an iron biosensor in practice, operational factors can affect its performance. Hence, Tran et al. (2015) investigated the effect of pH of the sample, buffer strength of the anolyte, surrounding temperature, and external resistance on the performance of the lithotrophic MFC-based biosensor [17]. Their experimental results showed that these parameters affect the performance of the lithotrophic MFC-based biosensor at different degrees.

#### 2.6.1. pH of the sample and buffer strength

Although it is proposed that the lithotrophic MFC-based biosensor is always operated in a manner such that the anolyte is buffered, pH of the sample can still slightly affect its performance. It was clear that samples with pH values falling in the range of 7–9 could lead to approximately 20% higher levels of the current generated by the device, in comparison with those with other pH values. In order to decrease material cost when applying the lithotrophic MFC-based biosensor in practice, the amount of anode buffer should be reduced. As reported by Tran et al. (2015), a 10-fold diluted buffer only reduced the generation of electricity by about 15%. Thus the effect of the buffer strength did not appear to be critical and reducing the amount of buffer used in practice is recommended.

For heterotrophic systems, similar effects of pH of the sample can be expected for buffered anolytes [49], but for field applications with real wastewaters, buffer strength of the anolyte can significantly affect the performance of the devices and should be carefully controlled [50]. Specifically, according to Gil et al. (2003), certain buffer strength is required to reduce the proton limitation in a MFC-based BOD sensor [49]. The fact that the performance of the lithotrophic MFC is less affected by buffer strength than pH is relatively striking. Possibly, the electrical current generated in this system is not great enough to be limited by proton transfer.

#### 2.6.2. Surrounding temperature

Understanding the effect of surrounding temperature on the performance of the lithotrophic MFC-based biosensor is also very important for practical applications. Surprisingly, the optimal range of surrounding temperature for the device to function is 30–40 °C, which is quite narrow. When operated at temperatures outside this range, the current generated by the MFC was 2- to 3-fold lower [17]. The narrow optimal range of temperature will likely limit the deployment of the equipment in reality.

Temperature also affects the performance of heterotrophic MFC-based biosensors in a similar manner. According to di Lorenzo (2009), MFC-typed BOD/COD sensors could give a higher current and a faster response when operated at temperatures higher than 30 °C. This temperature range was also considered optimal for a wastewater-fed single-chamber MFC, although decreasing the temperature from 32 °C to 20 °C did not have a significant effect on the performance of the MFC [51]. These similar responses to temperature in both heterotrophic and lithotrophic MFC systems is reasonable, as the MFCs are normally operated under ambient temperatures and thus mesophilic anode bacteria can be expected to be enriched.

#### 2.6.3. External resistance

External resistance is a key parameter limiting the performance of many MFC systems [14],[25]. In a lithotrophic MFC-based biosensor, the higher the external resistance, the lower the current that could be generated, but the relationship between these two parameters was not inversely linear [17]. The relationship is similar to those observed in other heterotrophic MFC systems, suggesting that external resistance can significantly affect the performance of the lithotrophic MFC-based biosensor. Furthermore, it has been shown that the magnitude of external resistance can also affect the response time and the recovery time of a MFC [52]. For instance, di Lorenzo et al. (2009) observed that the response time of a BOD and toxicity sensor could be reduced by 67% when the external resistance decreased from 500 Ω to 50 Ω [4]. Stein et al. (2012) also found that a lower external resistance resulted in a clearer and more sensitive response while a higher external resistance could help the MFC recover more quickly after change. Lithotrophic MFCs, however, always responded immediately (e.g. in less than 60 sec) to any change in the concentration of Fe^2+^ in the anolyte, regardless of the magnitude of the resistance being tested [17]. Thus, for this system, operators only need to select an external resistance that enables the generation of the highest current so that changes of the current are most apparent.

## 3. Challenges and propositions to overcome

It has been known that the performance of MFCs is limited by several factors, including the microbial activity, the electron transfer process, the internal resistance of the device, and particularly the cathode reaction rate [11],[14],[49],[53]. As evidenced, these limiting factors also affect the performance of the lithotrophic MFC-based biosensor. However, considering the application aim of using the device as a biosensor, it is the stability of the electrical signal that is required, not a high power output. Factors affecting the stability of the electrical signal can reasonably be identified as the microbial activity and the electron transfer process, rather than the other factors listed above. Therefore, in order to improve the performance of a lithotrophic MFC for use as an iron biosensor, microbial activity and the electron transfer should be addressed.

### 3.1. Selection of the inoculating sources

As mentioned above, in order to have a stable and well-performing microbial community that enables a stable electricity generation of the lithotrophic MFC, the selection of the microbial source for inoculation is critical. As suggested by Nguyen et al. (2015), inocula from natural sources which show clear indicators of iron metabolism can potentially provide a diverse and balanced collection of lithotrophic bacteria that can be enriched and function stably in a MFC [23]. Indeed, most well-performing and stable open-system MFCs are operated with mixed cultures enriched from natural microbial sources [37],[38]. In principle, this is a logical requirement, particularly for heterotrophic systems, as the diversity of the enriched microbial communities can handle the diversity of substrates to enable the performance stability of the systems. Thus, a diverse mixed culture in a lithotrophic MFC might be even better for its sensing performance. The enrichment and stabilization of a working community from a natural microbial source may require more time, but this is not considered as a critical matter in practice.

### 3.2. Performance stability

Even when the lithotrophic MFC is in steady state, its generated current may decrease after a significant time of operation (e.g. 12 months), although the response of the device to Fe^2+^ is unchanged, as mentioned earlier. In addition, the levels of the currents generated by different MFCs operated under the same conditions are not always equivalent. This is partially due to the effects of operational parameters, as discussed above. It should be recalled that different operational conditions affect the performance of the MFC at various degrees. Indeed, fluctuating performance was also observed with heterotrophic MFC-based sensors and a strictly controlled operation was recommended to reduce background [36]. Another potential factor affecting the long-term performance of the lithotrophic MFC-based biosensor can be insoluble ferric products precipitating on the anode surface and gradually hindering the electron transfer. Furthermore, in a long-term use of the system, electrochemically active heterotrophs may grow on organic contaminants in the sample and will likely alter the activity of the anode microbial consortium, as well as the electrical signal. The mentioned issues can be challenging if the sensing mechanism is based on the absolute value of the electrical current generated. For the lithotrophic MFC, one suggested solution to overcome this challenge is a calibration before using the device for any measurement [17].

Tran et al. (2015) also posed some propositions for improving the stability of the performance of the lithotrophic MFC-based biosensor. The first proposition is to replace the anode material with a material that can stimulate biofilm formation, such as graphite felt. The first-version system, operated with graphite granules as the anode material, seems to favor suspending bacteria that electrochemically function through self-produced mediators, and thus hardly achieved a steady performance due to anolyte wash-out or unstable bacterial density [17]. Graphite granules are known to have low porosities [54], which may lead to limited biofilm growth on them. Electrochemically active bacteria transferring electrons to the electrode via self-produced mediators have been reported to be associated solely with MFC systems operated with graphite granules as the anode material [45],[55]. Therefore, replacing graphite granules with graphite felts would likely result in less suspended electrochemically active bacteria and promote more biofilm formation in the anode. Other MFC systems operated with graphite felts as anode materials usually harbor biofilms formed on their anode surfaces [37],[56],[57]. Such a biofilm would ensure a stable microbial community that can be long-lasting and have a steady function [38]. Furthermore, the biofilm could be strengthened if the MFC is operated in continuous mode, which is the second proposition for improving the performance of the system. It is believed that a continuous mode might ensure the generation of a continuous current that is stable (much less affected by environmental factors than the batch-type current) and reflect the change of substrate concentration in the anolyte in a real-time manner [3]. Hence, the operation of the lithotrophic MFC in the continuous mode, combined with the use of graphite felt as its anode material, is expected to improve the stability of its performance and thus its iron sensing capability.

### 3.3. Limited detection range

While the detection range is not a matter to heterotrophic systems, it is a limitation of the first version of the lithotrophic MFC-based biosensor. The concentration detection range of the lithotrophic MFC for Fe^2+^ is 3–20 mM and that for Mn^2+^ is about 1–3 mM [17]. These narrow ranges might also limit the sensing capability of the device. Particularly, as the common concentration of Fe^2+^ in ground water samples falls below 3 mM, the lower detection limit of the MFC should be reduced. Further improvements are thus required and should focus on this matter. One possible improvement can be to reduce the volume of the anode chamber. Previous studies have shown that reducing the volume of the anode chamber significantly improved the sensitivity and detection limit of a BOD sensor [4]. Consequently, the volume of the anode chamber might be a cause for the high lower detection limit of the lithotrophic MFC-based biosensor for Fe^2+^. Therefore, experiments examining smaller volumes of the anode chamber should be conducted to investigate whether the detection range of the device can be expanded. In addition, as mentioned above, a sensor-type design can increase the response sensitivity and accuracy of MFC-typed biosensors and should be also applied for the lithotrophic MFC-based biosensor.

## 4. Opportunities

The people in rural areas in developing countries (such as Vietnam), having no access to public water supply and often use water from underground sources without being aware of its quality. For example, it was reported that of more than 16 millions people living on the Red River delta areas in northern Vietnam, 11 million have no access to clean water [58]. The water from underground sources can be contaminated with metals such as iron and manganese, which can cause several physiological malfunctions to the human body following exposure [59]. Populations in remote areas are in need of a method to quickly assess the presence of these toxic metals in their water; unfortunately, the current methods for the detection of the metals are mostly based on chemical tests that can be done only in laboratories or by using kits that are time-consuming, not environment-friendly, or not cost-effective. Hence, an on-site biosensor to detect metals such as iron and manganese in water sources would be contributive to a sustainable life of people in rural areas in developing countries.

Although still facing some challenges, as discussed by Tran et al. (2015), the potential application of the lithotrophic MFCs as metal biosensors is promising. As discussed earlier, the device can produce electrical currents only when ferrous iron is present and a linear correlation between the current and the concentration of Fe^2+^ could be applied within the concentration range of 3–20 mM (r^2^ = 0.98). Moreover, other possible electron donors cannot compete with ferrous iron at the same level. This specificity in detecting iron is attributed to the specific iron-oxidizing bacterial consortium enriched in the MFC [17]. Therefore, evidence that the device can detect iron in water samples (based on the appearance of electrical current) is considered reliable. The presence of ferrous iron will reflect the presence of iron in the samples, which in turn usually indicates the co-presence of other metals [58]. Thus, the detection of iron by the lithotrophic MFC-based biosensor can be also regarded as a warning about the presence of other metals in a water sample.

Simple handling is also an application advantage of the system. As mentioned previously, the MFC can be operated as an open-system biosensor as long as a suitable microbial community is enriched at the anode. As an open system, the device does not require special care or complicating renewing procedures. It is even more notable that the device can be operated with neutral-pH water samples, which reduces much of the work possibly required for pH adjustment if an acidic condition is needed. This is because the MFC is distinctively operated with neutrophilic iron bacteria, rather than the more popular acidophilic bacteria. Therefore, it is easier and more convenient for users to deploy the device in field applications.

Although the high [Fe^2+^] detection range can be considered a limitation of the MFC, it also implies that the device can be used to detect waters over-polluted with Fe. The device will be particularly helpful in quickly assessing any Fe or Fe^2+^ pollution situation. Quick assessment due to the quick response to Fe^2+^ of the lithotrophic MFC-based biosensor and its portability are also two other advantages of this system. Indeed, the small size of the device (only about 12 cm × 12 cm × 5 cm) [23] makes it portable. Portability and quick response suggest the prospect of innovating the device toward a portable quick-kit for the detection of iron in water samples. Commercial kits for quick detection of ferrous iron are available but they are costly (ca. 20USD/kit). The lithotrophic MFC-based biosensor can not only quickly sense the presence of iron but may also be reused unless the reactor is damaged, which making it more cost-efficient. A thorough comparison of lithotrophic MFC-based iron sensors with other technologies used for the same purpose is shown in Table 3.

**Table 3.** The comparison of the lithotrophic MFC-based biosensor to competing technologies.

Sensing platform	Specificity	Sensitivity & detection limits	Detection time	Portability	Handling	Cost	Reusability	Other opportunities
The lithotrophic MFC-based biosensor	Relatively high	Relatively high; detection range: 3–20 mM	Short (60 sec)	Yes	Simple; no special care	Low	Yes; for long (until the device is damaged)	Bioremediation and on-line monitoringof metals
Commercial quick detection kits	Very high	Very high; broad detection range	Short	Yes	Simple	High	No	No
Laboratory analytical tests	Very high	Very high; broad detection range	Long (time is required for sampling, sample processing and analysis)	No (samples should be brought to laboratory)	Not simple; skilled personnel required	High	No	No

The linear relationship between the Fe^2+^ concentration and the electrical signal produced by the lithotrophic MFC suggests that the device can be used as a biosensor not only to detect iron, but also to monitor the amount of iron. If the device can be operated in a continuous mode, real-time monitoring of the Fe^2+^ concentration will be even feasible. However, in order to realize those application potentials, several limitations need to be overcome, as already discussed.

An extended application potential of the lithotrophic MFC can be bioremediation of metals [23]. In fact, bioremediation using MFC and MFC-related systems has been reported by a number of publications [60]–[63]. In those reports, the bioremediation is based on exploiting the cathode reactions of the MFCs to reduce oxidized forms of metals. In contrast, the lithotrophic MFC can offer oxidation-based bioremediation. Potentially, the system could be used for an accelerated bioremediation of iron in water samples, as the soluble ferrous salts could be converted to insoluble ferric salts by the activity of the anode electroactive iron-oxidizing community.

## 5. Conclusions

Several MFC systems involving bacterial lithotrophy have been reported but only those operated with enriched iron-oxidizing bacteria can be considered fully lithotrophic systems that have the potential to be used as biosensors for iron or metals in general. The system is well established and characterized in terms of configuration, fabrication, and operational parameters. It shares some common features with the heterotrophic counterparts, but also has its own distinctive properties. The properties of an open-system, the operation at neutral pH, the relatively high specificity in detecting Fe^2+^, portability, and reusability are notable characteristics of the device and also the advantages that differentiate it from competing sensing technologies. However, in order to realize the practical application of the device, challenges to maintain a stable microbial community in the anode, a stable performance, and a broader detection range should be overcome by future research.
